# Establishment of a Stable β-Casein Protein-Secreted Laoshan Dairy Goat Mammary Epithelial Cell Line

**DOI:** 10.3389/fvets.2020.00501

**Published:** 2020-08-13

**Authors:** Hongyan Zhang, Tianzhen Liu, Boyu Li, Kang Zhang, Dong Wang, Ying Liu, Lijiang Ge, Yunliang Jiang, Feng Su

**Affiliations:** Shandong Provincial Key Laboratory of Animal Biotechnology and Disease Control and Prevention, College of Animal Science and Veterinary Medicine, Shandong Agricultural University, Tai'an, China

**Keywords:** immortalization, goat mammary epithelial cells, SV40T antigen, β-casein secretion, cells apoptosis

## Abstract

Mammary epithelial cells are widely used as models in mastitis research and as tools for mammalian bioreactors; however, the short lifespan of these cells limits their utility. Several mammal epithelial cell line models have been established; however, the secretion capacity and the bacterial sensitivity of these lines have not been effectively evaluated. In this study, a stable immortalized goat mammary epithelial cell (GMEC) line was constructed by transfection with the *SV40* gene. The monoclonal cells were then passaged through more than 50 generations after puromycin selection. The GMEC line was evaluated by reverse transcriptase polymerase chain reaction, the cell cycle, karyotype analysis, detection of apoptosis, Western blotting, and β-casein (CSN2) inducible assays. The GMEC line had a strong proliferation capacity relative to the primary GMECs. GMECs had the same karyotype as the primary cells. The GMEC lines maintained basic biological properties and had estrogen, prolactin, and progesterone receptors as same the primary cells. Additionally, the cells and the cell line could synthesize and secrete β-casein proteins. Finally, the rate of apoptosis of the transfected cells suggested that the cell line could provide a useful tool for signal research and mammary gland bioreactors.

## Introduction

Mastitis is characterized by inflammation of the mammary glands induced by mechanical damage and microbial infection and can be classified as subclinical or clinical mastitis (1). In general, injury of the mammary glands results in bacteriological changes in milk and pathological changes in glandular tissues (2, 3). Subclinical mastitis in ewes is often induced by bacterial infections (4). Release of cytokines caused by both local and systemic inflammatory reactions also triggers an inflammatory cascade—the “acute phase response” during inflammation—and leads to the acute phase of coliform mastitis (5, 6). Recent work suggests that the effects of mastitis are not only restricted to the udder but also can affect reproductive efficiency (7). Thus, there is a pressing need to find ways to reduce the incidence of mastitis through the production of antimicrobial peptides. Previous study reported production of antimicrobial peptides in mammary tissue is an effective way to reduce incidence of mastitis (7). This methods usually employed transgenic methods, which generally involve developing a cell model for antibacterial and secretion assays. Therefore, a stable cell model is necessary for gene editing of animal reproductive traits and the use of the mammary gland as a bioreactor.

Mammary epithelial cells (MECs) play a vital role in the onset of the innate immune response caused by goat mastitis (8). Toll-like receptors (TLRs) are the first immune barrier in the mammary gland. TLRs that attach to the surface of cells can recognize specific patterns in pathogens and release cytokines, contributing to the recruitment and activation of effector immune cells in the mammary gland. TLRs also play a critical role in the identification of microbial pathogens (9, 10). Additionally, the milk produced and secreted from MECs is under the control of hormones after delivery. For these reasons, MECs are often target cells for mammary bioreactors (11). Therefore, the establishment of immortalized goat mammary epithelial cells (GMECs) is important for reducing the incidence of mastitis and the production of antibacterial peptides.

There are currently two methods for developing cell lines. The first is through the use of human telomerase reverse transcriptase (hTERT). For example, to construct MEC lines, Previous study isolated GMECs from the mammary tissue of dairy goats and immortalized them with human telomerase reverse transcriptase (hTERT) (12, 13). However, some studies have shown that immortalization of normal cells by transfected hTERT has low transfection efficiency and can induce dysplasia and even crisis (13). Furthermore, hTERT-expressing cell lines may experience crisis after prolonged lifespans, and immortality is often not a consequence of hTERT expression in normal diploid cells (12, 13). The second method for developing cell lines is through the SV40 method. For example, some studies have shown that the expression of SV40 large T antigen (*SV40 LT*) genes in healthy cells can circumvent the cell cycle M1 (senescence) through the inhibition of the p53 and p16 pathways, thereby resulting in tumorigenesis and immortalization of cells (14–16). The efficiency of *SV40 LT* gene infection is significantly higher than that of hTERT (17).

Here, purified MECs were isolated from goat mammary tissue, and a cell line was established through a transfer plasmid containing the *SV40 LT* gene, followed by evaluation of its bioactivity over 50 generations compared with primary cells. The assays showed that these cells preserved the key characteristics of MECs and thus provide a robust model for mammary secretion assays and disease research.

## Materials and Methods

### Ethics Statement

The 2-year-old (45 days post-parturition) Laoshan dairy goats used in this study were bought from Zhengda Company from China (Taian, China) and accommodated in appropriate livestock housing and fed ad libitum. Goats were sacrificed with sodium barbital after anesthesia. All procedures involving animals were approved by the Animal Care and Use Committee of Shandong Agricultural University. Mammary tissue was collected after the goat was injected with sodium barbital (5 mg/kg) following anesthesia (subcutaneous injection).

### Reagents and Chemicals

DMEM/F-12 and FBS were purchased from BI (FBS, BI, Kibbutz, Israel). Insulin, hydrocortisone, and collagenase I were purchased from Sigma-Aldrich (St. Louis, MO, USA). Trypsin was purchased from Gibco (Gibco Lab., Grand Island, NY, USA), and ovine prolactin was purchased from SGMEC (Israel). Rabbit anti-keratin 18 (CK18), rabbit anti-progesterone receptor (PR), rabbit anti-estrogen receptor α (ERα), rabbit anti-prolactin receptor (PRLR), rabbit anti-β-casein (CSN2), and rabbit anti-cleaved caspase-3 were obtained from Abcam (Cambridge, UK).

### Isolation and Purification of Primary GMECs

MECs were derived from a mastitis-free Laoshan dairy goat that was in its lactation phase. Mammary tissue was obtained by a surgical operation after anesthesia by chloral hydrate. The tissue was then disinfected and cut into 1-mm^3^ cubes. The 1-mm^3^ tissues were then digested with 0.2% collagenase I at 37°C for 4 h, filtered through a 200-μm mesh screen and washed with phosphate-buffered saline (PBS) until the supernatant was free of turbidity. The cells were seeded into DMEM/F12 medium and incubated in an incubator at 37°C with 5% CO_2_. Then the cells were purified with differential digestion methods.

### Transfection of Cells With the *SV40 LT* Gene

The packaged virus which containing *SV40 LT* gene were obtained and concentrated by human 293T cells. In details, Cell supernatants were then harvested after co-transfection with pLVX-EGFP-T2A-Puro-SV40T (10 μg), psPAX2 (5 μg), and pCMV-VSV-G (7.5 μg) into human 293T cells (6 × 10^6^ cells/100-mm dish) for 48 h using the TransIntro^TM^ EL transfection reagent per the manufacturer's instructions. The cell supernatant medium was then filtered through a 0.45-μm membrane filter and mixed with 60% 5 × PEG8000 at 4°C overnight. The supernatant was discarded after centrifugation at 4,000 g for 20 min, and the tube was left to settle for 2 min. Finally, the residual liquid was removed, and an appropriate amount of lentivirus solution was added to dissolve the lentivirus virus. The purified lentivirus was used for the GMEC transfection procedures in the presence of 0.5 μg/ml of polybrene. Isolated primary GMECs were cultured in 100-mm culture-grade plastic dishes for 12 h before transfection with the virus that containing *SV40* LT gene. Then the virus were added into cells medium for cells infection. After 4 days of incubation, the cells were cultured with growth medium containing 1 μg/ml puromycin to select resistant cells.

### Cell Cycle and Growth Curve of GMECs

The cell cycle was evaluated by flow cytometry. Primary GMEC and GMEC lines (50 passages) were harvested and washed twice with cold PBS, followed by resuspension in cold 75% ethanol and fixation overnight at 4°C. The cells were then stained with PI/RNase Staining Buffer (BD, USA) per the manufacturer's protocol after washing cells twice with cold PBS. Finally, the aforementioned cells were detected using a BD FACSCalibur^TM^ flow cytometer. Primary GMEC and GMEC lines were seeded in 96-well-plates (cell number 1,000/well) for 7 days. The number of cells was counted every day.

### Cell Immunofluorescence

The GMEC line and primary GMECs were seeded on slides of cells. GMECs were rinsed with PBS, fixed by cell immunofluorescent fixative, permeated with 0.5% Triton X-100 and blocked with 5% FBS. Primary rabbit anti-CK18 (1:100), anti-ERα (1:100), anti-PR (1:100), and anti-PRLR (1:200) were incubated with cells overnight at 4°C. The coverslips were then washed three times with PBS. Alexa Fluor 555-labeled donkey anti-rabbit IgG secondary antibodies (H+L) (1:500) were added to the coverslips and incubated for 50 min in the dark. The coverslips were washed three times with PBS. Hoechst 33342 was added to the coverslips and incubated for 15 min at 37°C. The coverslips were again washed three times with PBS. Finally, the stained cells were visualized with confocal microscopy.

### Karyotype Analysis

Primary GMECs and GMEC-line (60 passages) were incubated into DMEM/F12 medium with 10% FBS for 12 h. Colchicine (1 μg/ml) was then added to the cells 5 h after the cells were overfilled. Next, the cells were digested and resuspended in 10 mL of 0.075 M KCl and incubated at 37°C for 25 min. The pre-cooled fixing solution (methanol:glacial acetic acid, 1:3, v/v) was added for pre-fixation. Finally, cells were dropped onto ice-cold glass slides and stained with PI for 10 min. The cells were examined by confocal microscopy after washing with PBS three times.

### RT-PCR Assays

Primary GMECs and GMEC-line cells (50 passages) were incubated in DMEM/F12 medium for 12 h. Total RNA was extracted using the TRIzol reagent (Invitrogen) and reverse transcribed into cDNA using the PrimeScript^TM^ RT Reagent Kit per the manufacturer's protocol. Finally, PCR amplification of the genes of interest was performed. The amplification primers of the *GAPDH, ACACA*, and *CSN2* genes were designed following the general PCR primer design principle and were synthesized by Sangon Biotech (Shanghai) Co., Ltd. PCR amplification was conducted in 25-μL reaction systems (Table 1).

**Table 1:** Primer pairs used in this study.

**Prime name**	**Primer sequence**	**Reactive condition PCR**	**Length/bp**
*SV40 LT*	F: AGTGGCTGGGCTGTTCTTTT	95°C for 5 min; s 94°C 30 s, 60°C 30 s	671
	R: ATGGGAGCAGTGGTGGAATG	72°C 30 s, 34 cycle; 72°C 10 min	
*ACACA*	F: GGTCAGCTCAGACACGCTCT	95°C for 5 min; s 94°C 30 s, 55°C 30 s	838
	R: GTTAGGGAAGTCATCCGCGT	72°C 30 s, 34 cycle; 72°C 10 min	
*CSN2*	F: ACAGCCTCCCACAAAACATCC	95°C for 5 min; s 94°C 30 s, 52°C 30 s	300
	R: TGAGAAAGGGACAGCCACGGA	72°C 30 s, 34 cycle; 72°C 10 min	
*GAPDH*	F: AAGTTCCACGGCACAGTCA	95°C for 5 min; s 94°C 30 s, 56°C 30 s	473
	R: GGTTCACGCCCATCACAA	72°C 30 s, 34 cycle; 72°C 10 min	

### Inducible Expression of CSN2 in GMECs

GMECs and goat fibroblasts were incubated in DMEM/F12 medium with 10% FBS for 12 h. The cell medium was then changed with serum-free induced medium (DMEM/F-12, 5 μg/mL insulin, 5 μg/mL prolactin, 10 ng/ml Epidermal Growth Factor (EGF) and 1 μg /mL hydrocortisone after the cells reached 80% confluency). The cell medium was then harvested after the cells had been induced for 48 h. The supernatant was collected after freeze-drying for 12 h. The proteins were then detected with the SDS-PAGE assay.

### Western Blotting

Primary GMECs and GMEC-line cells were collected and lysed in RIPA buffer containing PMSF (1 mM) at 4°C for 30 min, followed by centrifugation at 12,000 g for 10 min to collect the supernatant. Proteins were separated by SDS-PAGE and transferred to PVDF membranes. Membranes were blocked with 5% skim milk powder for 90 min and incubated with anti-ERα (1:100, Abcam), anti-PR (1:1,000, Abcam), anti-PRLR (1:300, Abcam), anti-cleaved caspase 3 (1:1,000, Abcam), anti-CSN2 (1:1,000, Abcam), and anti-GAPDH (1:10,000, Abcam) polyclonal antibodies overnight at 4°C. After three washes with TBST, the membrane was incubated with goat anti-rabbit IgG (H&L) secondary antibodies for 50 min at room temperature, followed by another three washes with TBST. Finally, the membrane was covered with BeyoECL Moon ECL Kit (Beyotime), and its chemiluminescence was detected using a chemiluminescent detector.

### Cell Apoptosis Assays

GMEC-line cells were collected after infection with lipopolysaccharide (LPS) for 48 h. The cells were then harvested and stained with a BD Pharmingen™ FITC Annexin V Apoptosis Detection Kit per the manufacturer's protocol. The cells were detected with flow cytometry.

### Statistical Analysis

Data from RT-PCR, cell proliferation analysis, and cell cycle, and CFU count assays were analyzed using SPASS software (SPSS, Chicago, IL, USA). Fold changes in target gene expression were presented as means ± SEM and were compared using one-way ANOVAs followed by Newman–Keuls tests. *P* < 0.05 were considered statistically significant.

## Results

### Isolation and Identification of Primary GMECs

**Figure 1:**
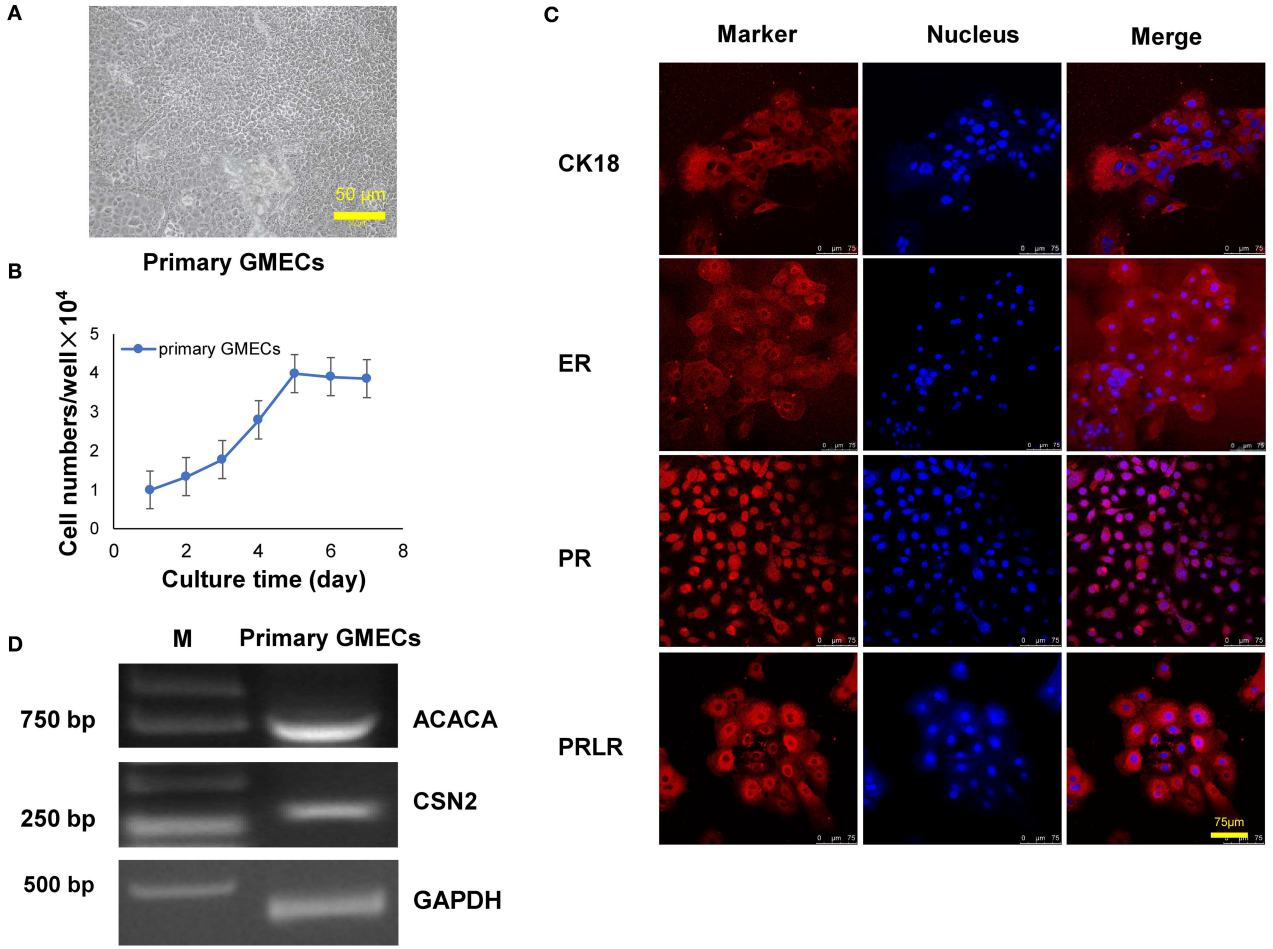
Characteristics of primary goat mammary epithelial cells (GMECs). **(A)** Primary GMECs observed under a light microscope (100×). **(B)** Growth curve of primary GMECs that was constructed from cell counts made on different days. **(C)** The immunofluorescence assays of primary GMECs for cytokeratin CK18, ERα, PR, and PRLR. **(D)** RT-PCR analysis of *ACACA* and *CSN2* in primary GMECs.

Primary GMECs were isolated from mammary gland tissue and seeded into Petri dishes. Figure 1A shows the purified MECs. GMECs grew in the medium, and the shape of the cells resembled paving stones (Figure 1A). Cell proliferation capacity was then assessed by its growth curve. Primary cells exhibited a steady pattern of growth similar to normal cells (Figure 1B). Cell markers were detected by immunofluorescence assays and the red signal exhibited by CK18, ER, PR, and PRLR for antibody-positive cells (Figure 1C). Primary cells were then assessed by RT-PCR assays. The band in Figure 1D indicated that the cells could express the *CSN2* and *ACACA* genes.

### Establishment of the GMEC Line

**Figure 2:**
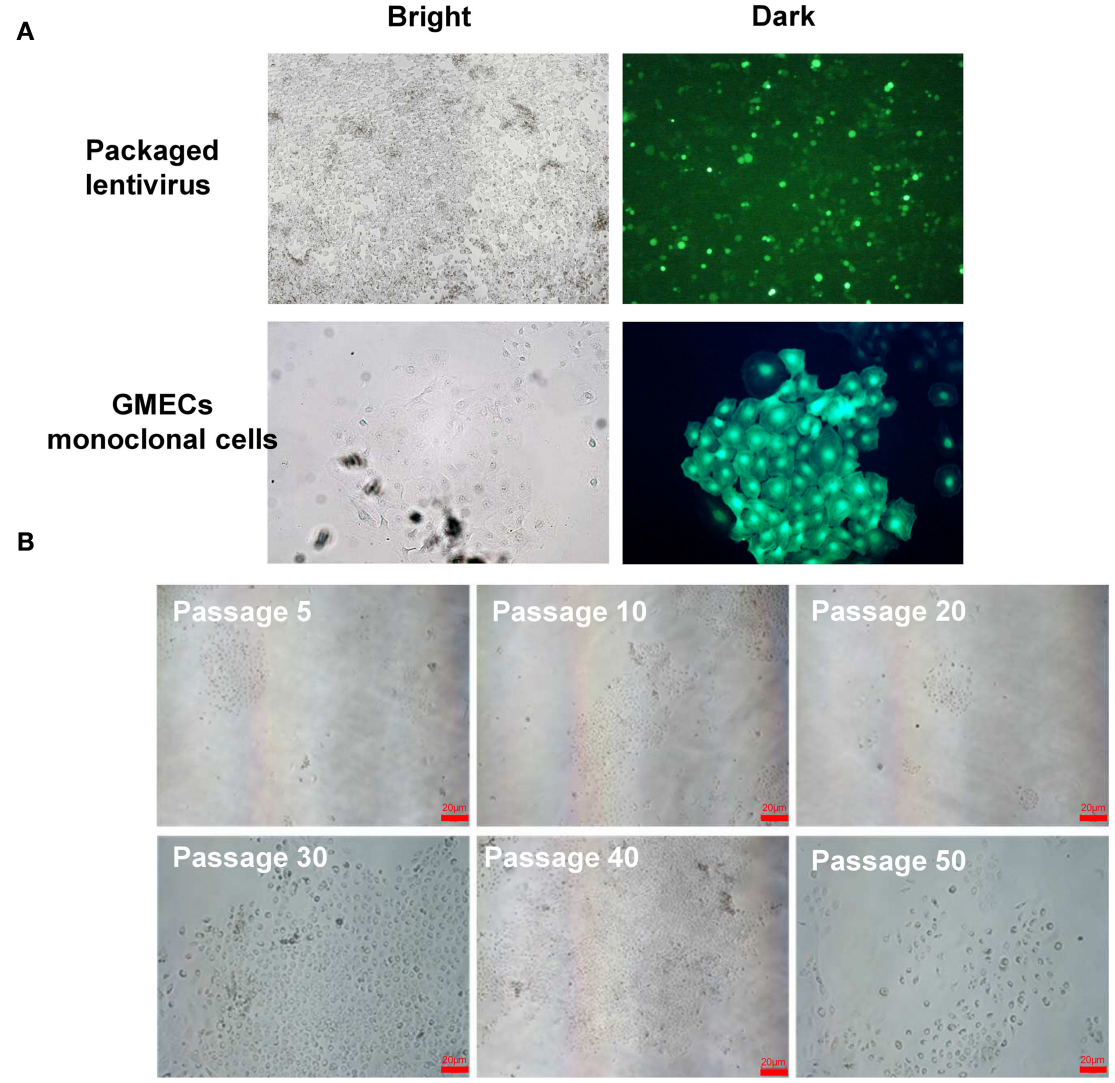
Lentivirus packaging and monoclonal selection of GMECs. **(A)** The process of packaging lentiviral particles of plasmids containing the *SV40 LT* gene. Image of 293T cells with the transfer plasmid containing the *SV40* gene (top). Monoclonal GMECs obtained after puromycin selection observed through a microscope (bottom). **(B)** Cellular morphology of the GMEC line at 5, 10, 20, 30, 40, and 50 passages. No morphological differences were observed.

The GMEC line was obtained by a transfer plasmid containing *SV40 LT* genes. Purified high-titer lentiviral particles were obtained after co-transfection with pLVX-EGFP-T2APuro-SV40 vector and pCMV-VSV-G and psMAX2 vectors in 293 T cells (Figure 2A). Monoclonal GMECs were selected by puromycin and were obtained after co-incubation with high lentiviral particles for 7 days (Figure 2A). Cells were then obtained from a single clone and subsequent passage from one generation to more than 60 passages. The shape of the cells resembled paving stones similar to the primary cells in different passages (Figure 2B). The green fluorescence of the GMEC-line cells ultimately disappeared.

### The GMEC Line Sustained Its Biological Characteristics

**Figure 3:**
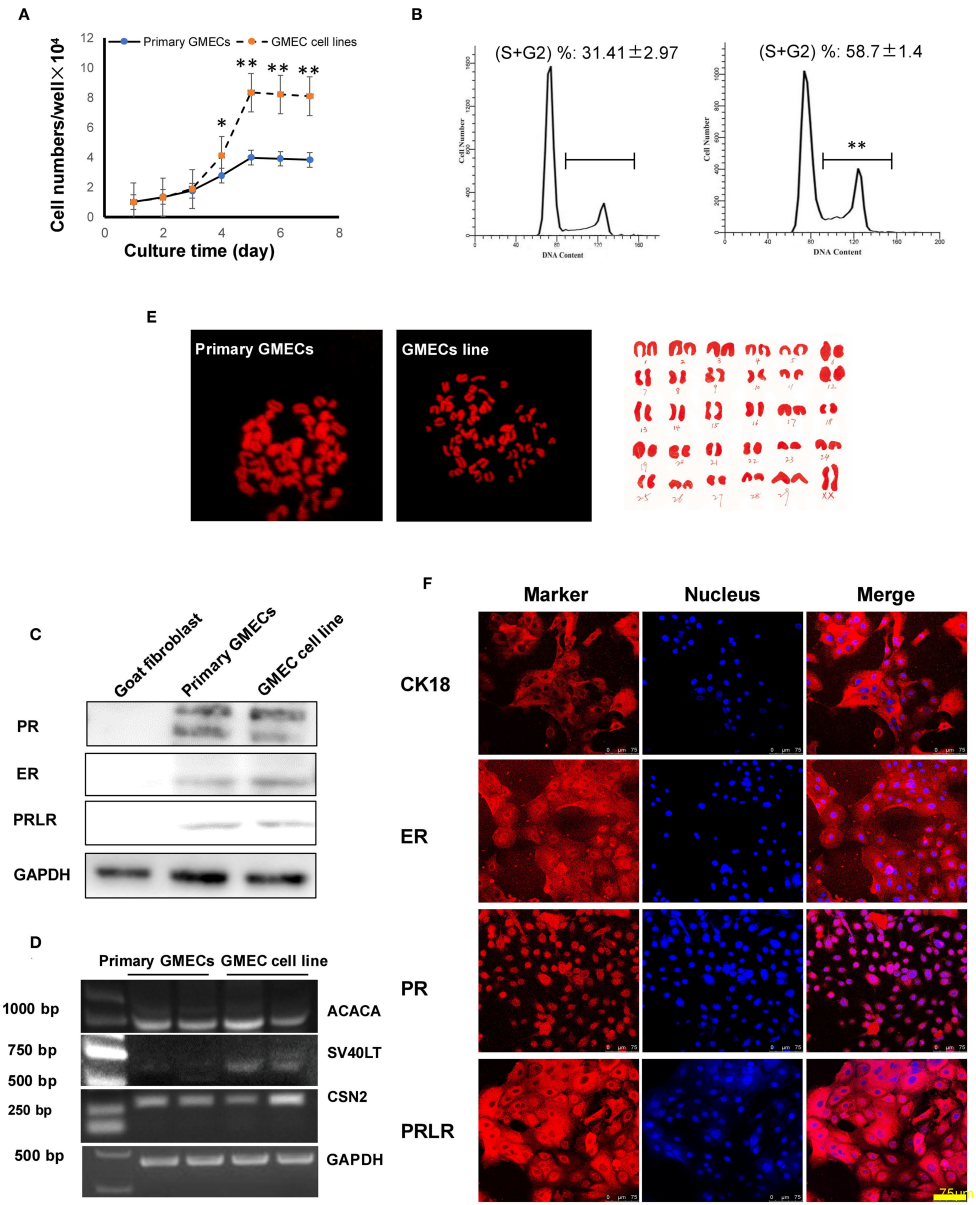
Characteristics of the GMEC line. **(A)** Comparison of the proliferation ability between the GMEC line and primary GMECs. (*p < 0.05). **(B)** Comparison of the cell cycle between primary GMECs and the GMEC line. **(C)** Western blotting analysis of ERα, PR, and PRLR. Expression of ERα, PR, and PRLR proteins was observed in both primary GMECs and the GMEC line. **(D)** RT-PCR assays of *SV40T CSN2* and *ACACA* mRNA. *SV40T* was detected in the GMEC line but not in primary GMECs. **(E)** Chromosome counts were evaluated by karyotype analysis in the GMEC line. **(F)** Immunofluorescence images of the GMEC line detected by cytokeratin CK18, ERα, PR, and PRLR, confirming its mammary epithelial attributes.

The GMEC line proliferation capacity was evaluated. The proliferation rate of the GMEC line increased significantly compared with primary GMECs after 4 days (*p* < 0.05) (Figure 3A). The cell cycle assay showed that the GMEC line had longer G1 and S phases compared with the primary GMECs (*p* < 0.05; Figure 3B). The bioactivity of the GMEC line was then assessed by Western blotting and RT-PCR. The Western blotting assay showed that the cell line retained PR, ERα, and PRLR similar to primary GMECs (Figure 3C). RT-PCR assays indicated that the cells could express *ACACA* and *CSN2* mRNA. However, *SV40 LT* mRNA was only detected in the GMEC line and not in primary cells (Figure 3D). The karyotypes of the cell lines were then evaluated. Both the primary cells and the GMEC lines had 30 pairs of chromosomes (2*n* = 60; Figure 3E). Finally, cell line markers were assessed by immunofluorescence assays. The red image shown in Figure 3F shows that the cell line retained CK-18, ERα, PR, and PRLR.

### Secretion of CSN2 Protein in the GMEC Line

**Figure 4:**
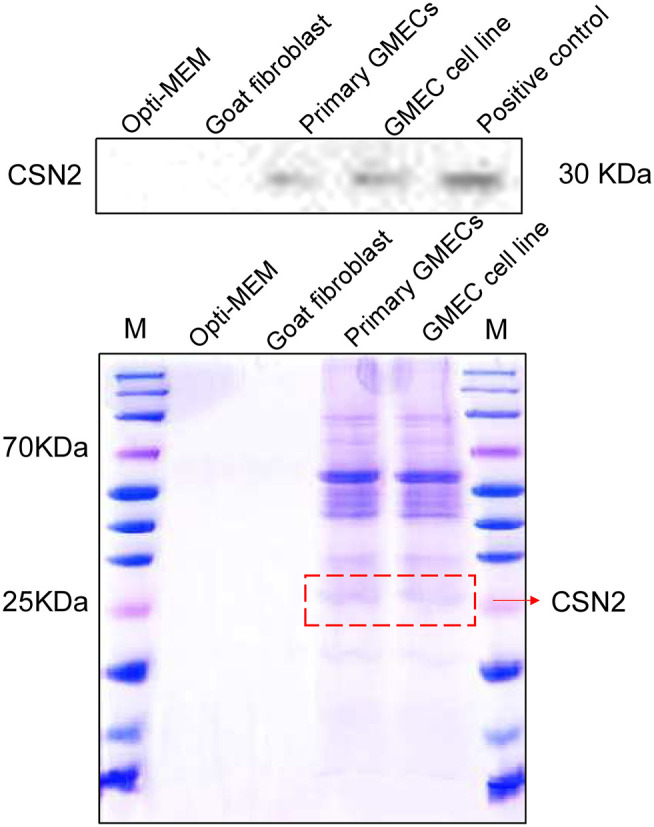
Inducible expression of casein protein. SDS-PAGE and Western blotting analysis of the CSN2 protein. Inducible expression of CSN2 detected by SDS-PAGE in primary GMECs and the GMEC line. Western blotting analysis of inducible-expressed CSN2 protein.

The secretion properties of cells were assessed by their inducible expression capacity. Secretion of the CSN2 protein was tested by SDS-PAGE and Western blotting analysis. The expression of CSN2 and other proteins is shown in Figure 4. The expression of these proteins was not detected in the goat fibroblasts cells and the negative control (Figure 4).

### The GMEC Line Is Sensitive to LPS

**Figure 5:**
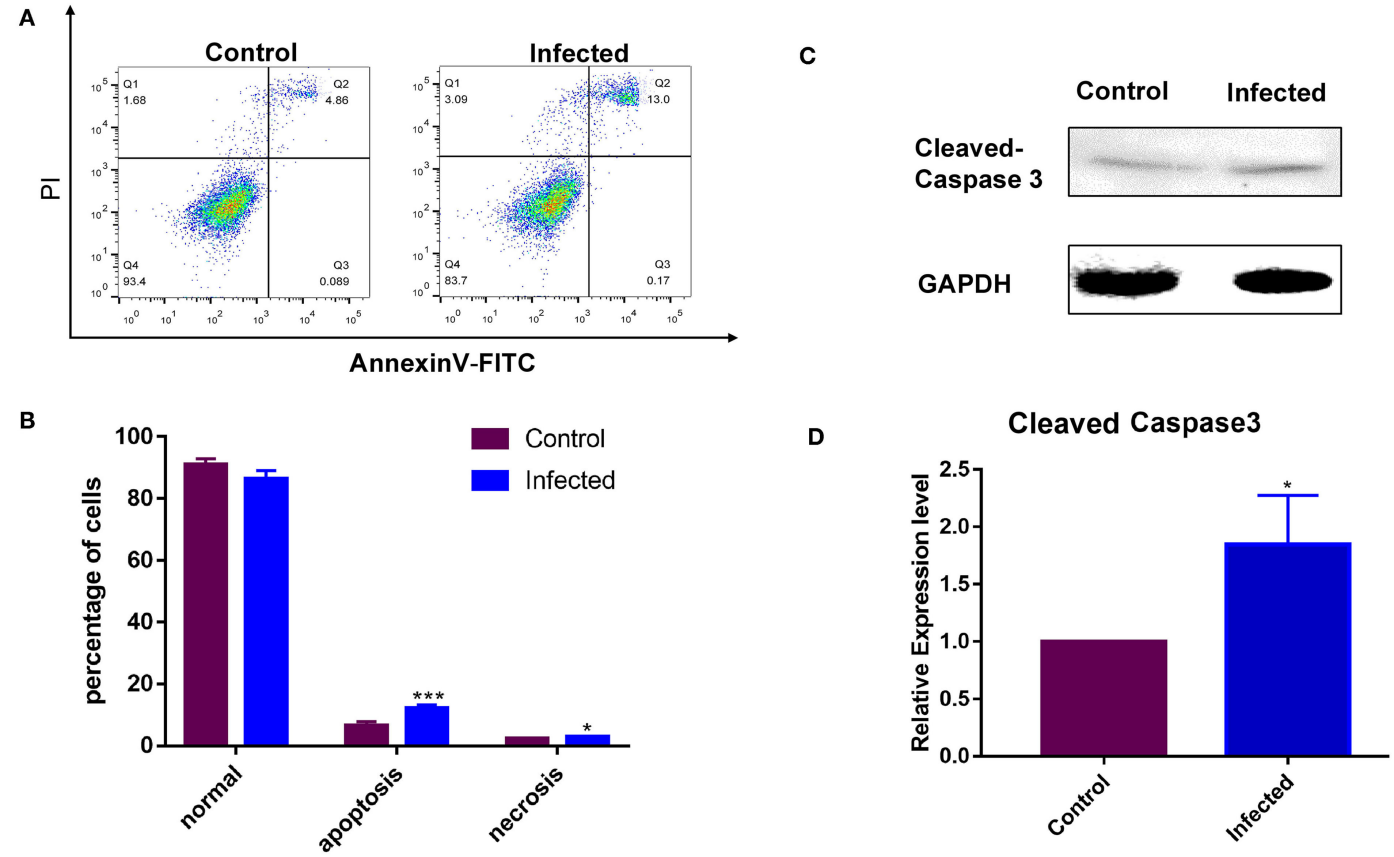
Susceptibility analysis of the GMEC-line cells activated by LPS. **(A)** Rate of cell apoptosis in normal GMEC-line and LPS-infected GMEC-line cells. **(B)** Significant differences were observed between normal and apoptotic cells after infection (*p < 0.05). **(C)** Western blotting analysis of cleaved caspase-3. **(D)** Significant differences were observed between normal and apoptotic cells after LPS stimulation (**p < 0.01).

The rate of apoptosis was used to estimate the susceptibility of the cell line to LPS. Non-infected GMEC line as a control group. A Western blotting analysis was conducted following incubation with LPS for 24 h. The LPS sensitivity of the GMEC line was estimated as the rate of apoptosis of GMECs. The rate of apoptosis of GMEC lines was much higher than that of the control (*p* < 0.001; Figures 5A,B). The expression of cleaved caspase 3 protein in GMEC lines was stronger than that of the control (*p* < 0.01; Figures 5C,D).

## Discussion

Mastitis is an important disease that causes substantial losses of dairy goats in the goat industry. Specifically, mastitis results in reductions in milk production and reproductive failure (18, 19). Generally, treatments with antibiotics provide an important method for preventing the occurrence of mastitis; however, this approach generates much environmental pollution (20). The development of gene-editing and transgenic strategies provides alternative, more environmentally friendly approaches for reducing the morbidity of mastitis. Furthermore, mammary bioreactors provide an effective means for building a protective barrier to resist bacterial infection, especially during the secretion phase (11). Thus, the establishment of a MEC line with stable secretion properties is important for mastitis research and the development of mastitis-resistant dairy goats. Here, we built a GMEC line that retained the bioactivity of MECs. These cells also retained their capacity for secretion and their sensitivity to LPS, confirming that this cell line could be used as a future tool for breast bioreactors and mastitis research.

Primary GMECs can be obtained via two methods: the tissue-block method or by digestion. Highly purified GMECs are more easily obtained by digestion relative to the tissue-block method. In this study, primary purified GMECs were taken from a Laoshan dairy goat by digesting mammary gland tissue pieces with collagenase I. This method differs from that of Shi et al. (21), as they obtained MECs by cultured tissue blocks. However, their method required more than 6 days; in addition, the purity of the cells was lower than that obtained by the digestion method (22, 23). Cell bioactivity was assessed by CK18, ER, PR, and PRLR proteins. Previous studies have reported that CK18 is an epithelial cell-specific protein marker; thus, the positive images of CK18 in GMECs indicated that the cells that we obtained were, in fact, epithelial cells (21). We also detected the expression of ER, PR, and PRLR in primary GMECs, which is consistent with a previous study (24). Previous studies have reported that the estrogen, progesterone, and prolactin receptors are crucial for mammary development. The prolactin receptor is especially important, as a previous study suggested that prolactin could promote mammary development via activation of the Jak2/Stat pathway (25). The *ACACA* and *CSN2* genes are also recognized as important for controlling the contents of sheep milk. Both of these genes were evaluated by RT-PCR, and mRNAs in the cell lines indicated that GMECs retained the proteins encoded by these transcripts as has been previously shown (24). The aforementioned evidence indicates that these monolayer cells retain their functional proteins and that the cell line consisted of MECs.

The lentiviral system provides an efficient means for transfection to mediate the infection of non-dividing cells. This system also permits large genetic payloads and maintains stable long-term transgene expression (26). The *SV40 LT* could induce the immortalization of cells by promoting the bypassing of the M1 phase of the cell cycle (senescence) (16), it is also binding the retinoblastoma protein (Rb) and the tumor suppressor protein p53 and finally inhibition cell lines transform into primary one. Potential tumorigenesis of SV40 LT mainly reflected in the binding ability to p53, In other words, although the tumor generation probability is very low, SV40 LT gene has potential capacity causing tumorigenesis (16, 26). In this study, the *SV40 LT* gene was packaged into a lentivirus system by a three-plasmid system and infected into primary GMECs. After selection by puromycin, the monoclonal cells were successfully obtained and amplified. Loss of GFP expression in cell lines suggested that the GFP mRNA was degraded in the cytoplasm, consistent with the findings of a previous study (18).

The bioactivity of the cell line was evaluated, and the cell line retained its proliferation capacity in addition to the expression of CK18, ER, PR, and PRLR as in primary MECs. The expression of ERα, PR, and PRLR was also demonstrated by the Western blotting analysis. Moreover, the *ACACA* and *CSN2* genes were expressed in the GMEC line as well as in primary GMECs; however, the *SV40 LT* gene was only expressed in the GMEC line. Karyotypic characterization also demonstrated the stability of the GMECs. Thirty pairs of chromosomes were detected from the GMEC line in contrast to the number of pairs of chromosomes that has been reported by previous studies. For example, *SV40 LT*-transfected cells have been reported to have chromosomal abnormalities, such as chromosomal duplications (27). However, the karyotype analysis in our current study indicated that our GMEC line had normal numbers of chromosomes.

MECs are the cells involved in lipid synthesis, storage, and milk secretion, and their secretion ability is controlled by a series of hormones, such as prolactin and insulin (28). CSN2 is the main protein secreted from the breast and has a molecular weight between 20 to 30 kDa (29, 30). In our study, CSN2 was detected in the primary GMECs and cell lines similar to previous studies. The application of prolactin and insulin is key for CSN2 secretion. In previous assays, Wang et al. (31) reported that prolactin was necessary for casein synthesis in the lactating mammary glands of goats. Another study showed that insulin can stimulate and regulate milk protein synthesis in GMECs (32) and can also play an essential role in the accumulation of mRNA in mouse MECs (31). Our results indicated that both prolactin and insulin are essential for CSN2 secretion.

The susceptibility of GMECs is important for the construction of cell models. LPS was used as an inducer in this study for its immunological stimulation and toxic pathophysiology. Some studies have indicated that primary GMECs are often activated by LPS and used as research models for mastitis (33). For this reason, the reaction of the cell line was evaluated after treatment with LPS. Activation of the TLR4 signaling cascade by LPS promoted the transcription of inflammatory cytokines, which eventually led to cell apoptosis and death (34). Caspase-3 plays a key role in the fragmentation of DNA and inducing morphological changes associated with apoptosis (35). In our assay, cleaved caspase-3 was significantly increased after the GMEC line was treated with LPS, indicating that the GMEC line was susceptible to LPS.

## Conclusions

In sum, a MEC line from Laoshan dairy goats was established via a transfer plasmid containing the *SV40 LT* gene. The bioactivity of the GMEC line was evaluated, and the cells retained ER, PRLR, and PR proteins; the cells also retained their capacity for secretion. The LPS stimulation assay indicated that the cell line provided a robust model for mastitis research and that it could also be used for mammary gland bioreactors.

## Data Availability Statement

The raw data supporting the conclusions of this article will be made available by the authors, without undue reservation.

## Ethics Statement

The animal study was reviewed and approved by Animal Care and Use Committee of Shandong Agricultural University.

## Author Contributions

YJ, HZ, and FS designed the experiments and drafted the manuscript. HZ, TL, KZ, DW, and YL carried out the animal care, sample collection, and performed the experiments. BL and HZ performed the data processing and biological information analysis. FS and LG interpreted the data. HZ and FS conceived the study and wrote the manuscript. All authors read and approved the final draft.

## Conflict of Interest

The authors declare that the research was conducted in the absence of any commercial or financial relationships that could be construed as a potential conflict of interest.
